# Piezoresistive Sensors Based on Electrospun Mats Modified by 2D Ti_3_C_2_T_x_ MXene

**DOI:** 10.3390/s19204589

**Published:** 2019-10-22

**Authors:** Patrik Sobolčiak, Aisha Tanvir, Kishor Kumar Sadasivuni, Igor Krupa

**Affiliations:** Center for Advanced Materials, Qatar University, Doha 2713, Qatar

**Keywords:** pressure sensor, copolyamide, MXene, electrospun nanofibers

## Abstract

The preparation methodology and properties of electroconductive, electrospun mats composed of copolyamide 6,10 and Ti_3_C_2_T_x_ are described in this paper. Mats of several compositions were prepared from a solution of n-propanol. The obtained electrospun mats were then tested as piezoresistive sensors. The relative resistance (A_R_) of the sensor increased with an increase in the Ti_3_C_2_T_x_ content, and materials with relatively higher electrical conductivity displayed noticeably higher sensitivity to applied pressure. The pressure-induced changes in resistivity increased with an increment in the applied force.

## 1. Introduction

Stretchable and wearable strain-sensing devices are suitable for motion detection, biomedical monitoring, and human-machine interaction [1,2,3,4,5]. These pressure-type sensors are based on numerous electrophysical phenomena, such as piezoelectric [6], capacitive [7], and piezoresistive [8] reactions towards mechanical stretching. Piezoresistive sensors are highly favored due to their high sensitivity, fast response, ease of fabrication, and low energy requirement. They are generally fabricated using a suitable polymeric matrix and electrically conductive fillers, such as graphite, graphene, or carbon nanotubes.

Recently, a new family of 2D materials called MXenes has been discovered and explored for various kinds of applications [9]. MXenes are chemically etched metal carbides and carbonitrides with the general formula M_n+1_X_n_, where n = 1, 2, or 3, and are derived from MAX phases, where M is an early transition metal; A is an A-group element, mostly IIIA and IVA, or groups 13 and 14; and X is either carbon and/or nitrogen [10]. Lately, MXenes have been used for the manufacture of various polymer-based sensors. For instance, Ma et al. [11] fabricated an extremely flexible and sensitive piezoresistive sensor based on MXene that showcased high sensitivity and fast responses while detecting subtle human bending-release activities and any other weak pressures.

A Ti_3_C_2_T_x_ MXene nanocomposite with single-walled carbon nanotubes equipped through a layer-by-layer spray-coating technique was utilized as a strain sensor with a detection limit as low as 0.1% [12].

Sponge-type sensors based on MXene have been industrialized; they exhibit high sensitivity for a broad pressure range, a low detection limit of 9 Pa, a rapid response time of 138 ms, and an outstanding durability of over 10,000 cycles [13].

Piezoresistive sensors centered on ultralight and superelastic aerogels were fabricated from MXene and reduced graphene oxide, employing their pressure-sensitive characteristics. The sensor showed extremely high sensitivity (22.56 kPa^−1^) and a fast response time (<200 ms) [14].

A bioinspired sensor produced by the amalgamation of Ti_3_C_2_T_x_, silver nanowire, and poly(dopamine)/Ni^2+^ generated a gauge factor >200 over a range of working strains up to 83% [15].

We have previously reported two studies on incorporating MXene into electrospun nanofibers. Ti_3_C_2_T_x_ and/or nanocellulose-reinforced polyvinyl alcohol (PVA) nanofibers were fabricated using electrospinning. PVA nanofibers containing 0.14 wt.% Ti_3_C_2_T_x_ exhibited a DC conductivity of 0.8 mS/cm, which is superior to that of similar composites previously reported [10].

A similar study carried out by Shao et al. [16] reported the production of PET/MXene nanofiber-based composites via electrospinning. Their study reported successful incorporation of MXene in polymer composite nanofibers for potential applications in flexible and wearable all-textile energy storage yarns.

In this study, we report the preparation, properties, and piezoresistive individualities of electroconductive, electrospun mats composed of copolyamide 6,10 and Ti_3_C_2_T_x_. We observed that the relative resistance (A_R_) of the sensor increased with an increase in the Ti_3_C_2_T_x_ content, and the materials with higher electrical conductivity showcased a significantly higher sensitivity to applied pressure. To the best of our knowledge, this is the first time that electrospun sensors based on 2D MXene have been prepared. The electrospinning approach exhibited several significant advantages, such as the ease of changing the size of the fibers by changing the electro spinning conditions. 

## 2. Experimental

### 2.1. Materials

Copolyamide 6,10 (Vestamelt X1010, EVONIK Industries, Marl, Germany), propanol (Sigma Aldrich, St. Louis, MO, USA), Ti_3_AlC_2_ (Y-Carbon, Ltd., Kiev, Ukraine), and hydrofluoric acid (Sigma Aldrich, St. Louis, MO, USA) were purchased.

### 2.2. Preparation of Ti_3_C_2_T_x_ Particles

Ti_3_C_2_T_x_ nanosheets were synthesized using the HF etching method described earlier [17]. The synthesized Ti_3_C_2_T_x_ was diluted in water and consequently delaminated by sonicating it in a bath sonicator for 60 min. The delaminated Ti_3_C_2_T_x_ dispersion was centrifuged at 3500 RPM for 45 min, and the supernatant, which consisted of delaminated Ti_3_C_2_T_x_ nano sheets, was then collected and dried. 

### 2.3. Characterizations

The surface morphology of the specimens was inspected using a field emission scanning electron microscope (FE-SEM, Nova Nano SEM 650, Hitachi, Japan). The SEM was equipped with an energy-dispersive X-ray spectroscope (EDS) that produces secondary electron images at a voltage of 3 kV and at various magnifications. All specimens were sputter-coated with 2 nm of gold before imaging with SEM.

Dielectric measurements were performed using a Novocontrol GmbH Concept 40 broadband dielectric spectrometer (Novocontrol Technologies GmbH & Co. KG, Montabaur, Germany). The data were collected over a frequency range of 0.1 Hz–3 MHz at a fixed temperature of 20 °C. Sample discs of 2 cm diameter were placed between two gold-coated copper electrodes of 2 cm diameter, and then were transported to the instrument for the process of data collection.

### 2.4. Pressure Sensor Performance

**Scheme 1:**
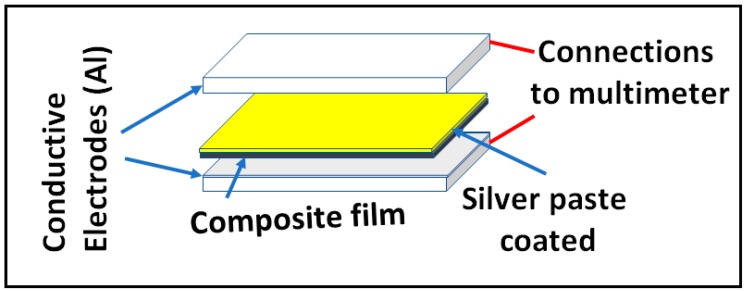
Schematic representation of the sensor layers and connections.

Schematic illustration of constructed sensors is shown in Scheme 1. In brief, composite films were covered in silver paste in order to improve electrical contact between the samples and electrodes. Conductive electrodes were attached to each side of the sample, and the electrodes were connected to a multimeter via copper wires. For the pressure sensor experiments, a shaker measurement rig was constructed using a signal generator and an electrodynamic shaker. The sample container was kept on the top of the shaker and was excited in the z-axis at peak amplitude.

In this linear domain, the piezo-resistive coefficient (also named gauge factor, *G*) was calculated using Equation (1):

(1) G=ΔRR0ΔLL0

where *Ar* = Δ*R/R*_0_ is the response amplitude or relative change in resistance, and *ε* = Δ*L*/*L*_0_ is the deformation of the sensor. 

## 3. Results and Discussion

### 3.1. Scanning Electron Microscopy

**Figure 1:**
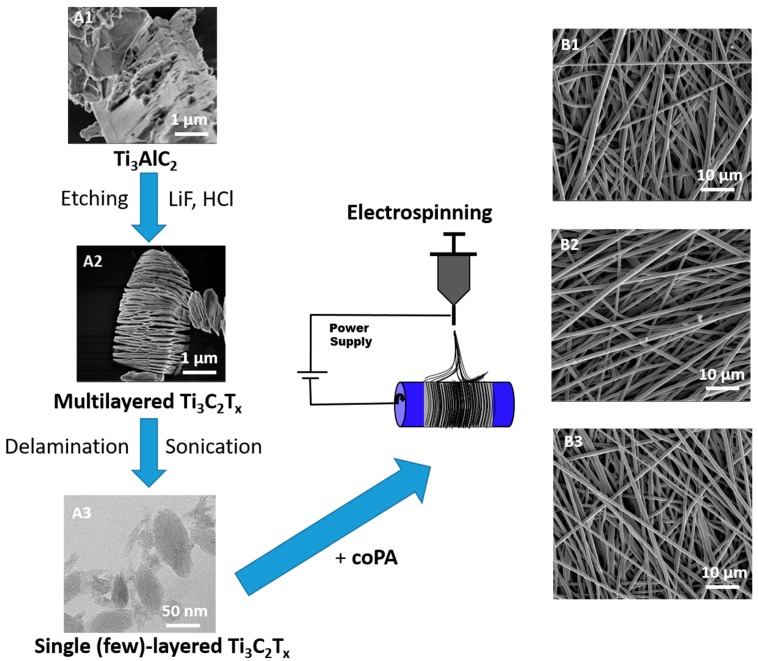
Preparation route of Ti_3_C_2_T_x_ and SEM images of precursor (A1), multilayered Ti_3_C_2_T_x_ (A2), and single (few)-layer Ti_3_C_2_T_x_. (B1) neat coPA, (B2) coPA and 2.5 wt.% of Ti_3_C_2_T_x_, and (B3) coPA and 5 wt.% of Ti_3_C_2_T_x_.

The fabricated delaminated Ti_3_C_2_T_x_ flakes were dispersed in propanol and mixed with a coPA solution to synthesize nanofibers via electrospinning. Figure 1 shows a schematic of the preparation process, starting from the multilayered Ti_3_C_2_T_x_ to the single-layer Ti_3_C_2_T_x_ nanosheets; the figure also shows images of the SEM and XRD analysis, which confirm the successful preparation of Ti_3_C_2_T_x_ through the process of etching and subsequent delamination.

Then, the Ti_3_C_2_T_x_ nanosheets were incorporated into coPA nanofibers via electrospinning and are shown as B1, B2, and B3 in the SEM images.

### 3.2. Electrical Conductivity

**Figure 2:**
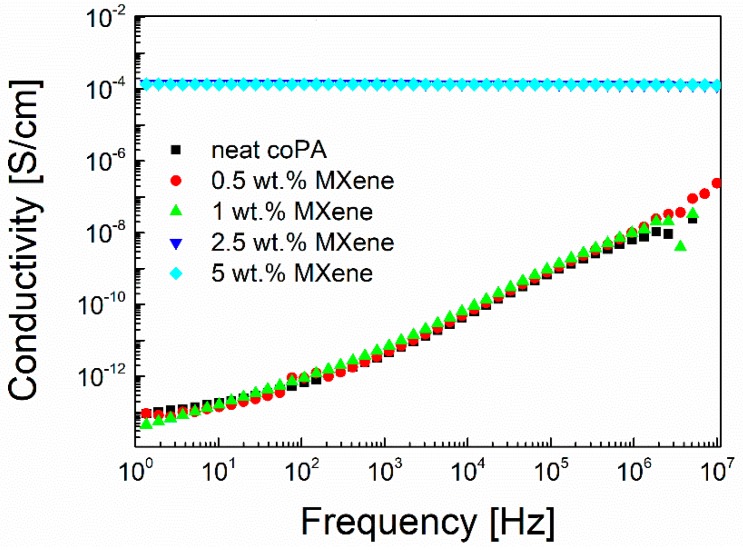
Electrical conductivity of electrospun coPA filled with Ti_3_C_2_T_x_.

The frequency dependence of the real part of the electrical conductivity of the electrospun mats at room temperature is shown in Figure 2. The electrical conductivity of the neat polymer and its composite filled with 0.5 and 1 wt.% Ti_3_C_2_T_x_ are comparable and indicate a nonconductive behavior. The electrical conductivity remarkably increased when the filler content increased to 2.5 wt.% Ti_3_C_2_T_x_, with the electrical conductivity increasing up to of eight orders of magnitude. The real part of the electrical conductivity is constant for the entire frequency range, demonstrating the conductive nature of the materials. A further increase in Ti_3_C_2_T_x_ content does not cause any noteworthy enhancement in the electrical conductivity.

### 3.3. Piezoresistive Touch Sensor

The piezoresistive effect is widely utilized in mechanical force sensors (pressure, acceleration, vibration, etc.). Piezoresistive sensitivity of a sensor is defined as the relative change in electrical resistivity due to applied mechanical strain [18,19]. Piezoresistive sensors based on polymer composites consist of a nonconductive polymer and a conductive filler. They require an optimal ratio of polymer/filler content to serve as high sensitivity touch sensors. When force is applied, the distance between the filler particles changes, resulting in a change in the resistivity of the sensor [20].

Prepared samples were electrospun structures that behaved like sponges. The gauge factor was calculated to be 4.5 at 25 °C. It is clear that the nanocomposite’s formulation led to improved sensitivity (*Ar* = ~50) and was exempt of noise at 100 N force.

The sample was subjected to a durability check by performing 100 cyclic experiments, and no noticeable change was observed in the sensitivity. 

**Figure 3:**
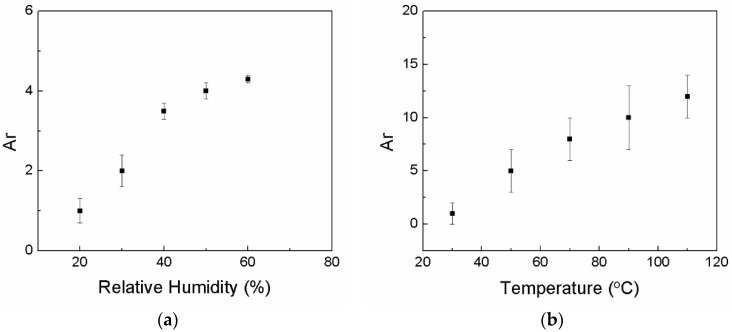
The effect of relative humidity (**a**) and temperature (**b**) on the sensor resistance.

Also, the effect on temperature and relative humidity on sensor performance was measured (Figure 3). 

To ensure accuracy of the measurement, five samples were measured. An error of ± 6.2 N at 100 N normal surface forces, temperature 25 °C, and a relative humidity of 32% was recorded.

Light finger tapping and finger shearing were identified using the newly manufactured pressure sensor (Supplementary Video 1). The setup was used to assess the pressure sensor’s response to light finger tapping (20 N force) with cycling experiments at altered loadings of Ti_3_C_2_T_x_, as shown in Figure 3a. The shell structure theory states that the resistivity of any conductive composite is affected by the application of external pressure, due to the formation and destruction of conductive networks. A similar mechanism has been published previously by utilizing copper in the case of carbon-like diamond composites [21] or in carbon nanotubes composites in a wide range of polymer composites materials [22]. In this study, the pressure sensitivity was heightened by using increasing amounts of fillers, which resulted in a large number of filler-filler and filler-polymer interactions. Ultrathin pressure sensors based on coPA/Ti_3_C_2_T_x_ composites with a thickness of 0.1 mm were equipped and tested. The relative resistance (A_R_) of the sensor was recorded (Equation (2)) with simultaneous application of force, which was applied in the range of 20–100 N.

(2) $$A_{R}=\frac{R-R_{0}}{R_{0}}$$

where *R* and *R*_0_ are the final (after applying a pressure) and initial resistance values of the sample, respectively.

**Figure 4:**
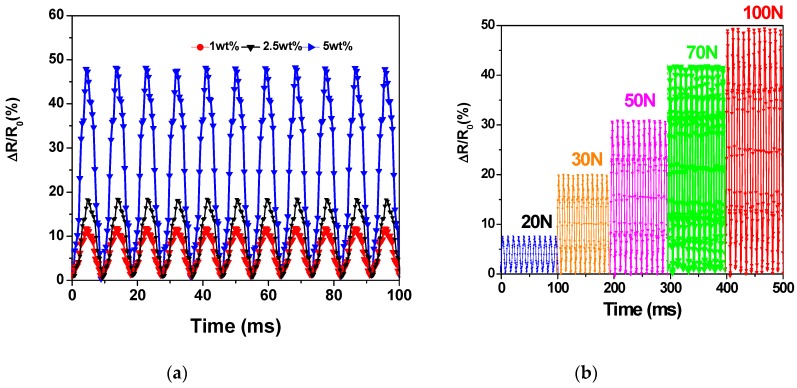
Pressure-induced resistivity changes for samples with various compositions of mats under a constant applied force (100 N) (**a**), and the pressure-induced resistivity changes at different forces (5 wt.% Ti_3_C_2_T_x_) (**b**).

The results are shown in Figure 4. With an increase in applied pressure, the relative resistance of the samples increased, which is the positive pressure coefficient of the resistance (Figure 4a). The electrical resistance of the Ti_3_C_2_T_x_-filled coPA electrospun mat recurrently alters with the uniaxial pressure due to the bulky number of links among Ti_3_C_2_T_x_ (nanoconfinement), indicating its potential to be used as a flexible force sensor. A video of the sensor in action can be seen in Supplementary Video 1.

## 4. Conclusions

Electrospun nanocomposite mats based on electroconductive, electrospun mats composed of copolyamide 6,10 and Ti_3_C_2_T_x_ were successfully prepared. The electrospun mats were tested as piezoresistive sensors, and their relative resistance (*A_R_*) was observed to increase with an increase in the Ti_3_C_2_T_x_ content. The high electrical conductivity of the materials resulted in a noticeably increased sensitivity to the applied pressure. Changes due to pressure-induced resistivity were also seen to have increased with an increase in the aplied force.

## Supplementary Materials

The following are available online at https://www.mdpi.com/1424-8220/19/20/4589/s1, Video S1: manuscript-supplementary.mp4.
